# Supplementary material for: Profiling the Typical Training Load of a Law Enforcement Recruit Class
Source: Int J Environ Res Public Health. 2022 Oct 18;19(20):13457. doi: 10.3390/ijerph192013457 (PMC9603164; doi:10.3390/ijerph192013457)
Supplement: Supplementary file 1 [file ijerph-19-13457-s001.zip › ijerph-1962988-supplementary.pdf]

Supplemental Table S1. Linear mixed effect model results.

Week	Comparison	Difference (m)	*p*-Value	Week	Comparison	Difference (m)	*p*-Value
1	2	9648.97	< 0.01	10	11	133.21	1
	3	9640.3	< 0.01		12	553.79	1
	4	11648.48	< 0.01		13	1831.76	1
	5	9689.17	< 0.01		14	3294.5	0.99
	6	10069.68	< 0.01		15	5027.52	0.8
	7	7511.02	0.95		16	1682.61	1
2	3	-8.67	1	11	12	-779.43	1
	4	1999.5	1		13	498.54	1
	5	40.2	1		14	1961.29	1
	6	420.71	1		15	349.39	0.99
	7	-2137.95	1		16	3694.3	1
	8	-4415.36	0.93		17	2722.69	0.99
3	4	2008.173	1	12	13	1277.97	1
	5	48.87	1		14	2740.72	0.99
	6	429.38	1		15	4473.73	0.92
	7	-2129.29	1		16	1128.82	1
	8	-4406.69	0.93		17	3502.12	0.99
	9	-2590.97	0.99		18	-4488.14	0.92
4	5	-1959.31	1	13	14	1462.74	1
	6	-1578.8	1		15	3195.76	0.99
	7	-4137.46	0.96		16	-149.15	1
	8	-6414.87	0.33		17	2224.15	1
	9	-4599.15	0.9		18	-5766.11	0.55
	10	-4934.92	0.82		19	-2685.52	0.99
5	6	380.51	1	14	15	1733.02	1

	7	-2178.15	1		16	-1611.9	1
	8	-4455.56	0.93		17	761.4	1
	9	-2639.84	0.99		18	-7228.85	1
	10	-2984.62	0.99		19	-4148.37	0.14
	11	-1651.4	1		20	-3458.58	0.96
6	7	-2558.66	1	15	16	-3344.91	0.99
	8	-4836.07	0.85		17	-971.611	1
	9	-3020.35	0.99		18	-8961.87	< 0.01
	10	-3365.13	0.99		19	-5881.38	0.51
	11	-2031.91	1		20	-5191.59	0.75
	12	-2811.34	0.99		21	-13163.96	< 0.01
7	8	-2277.41	1	16	17	2373.3	1
	9	-461.69	1		18	-5616.96	0.61
	10	-806.46	1		19	-2536.47	1
	11	526.75	1		20	-1846.68	1
	12	-252.68	1		21	-9819.05	< 0.01
	13	1025.29	1		22	-9693.09	< 0.01
8	9	1815.72	1	17	18	-7990.26	0.049
	10	1470.94	1		19	-4909.77	0.83
	11	2804.16	0.99		20	-4219.98	0.96
	12	2024.73	1		21	-12192.35	< 0.01
	13	3302.7	0.99		22	-12066.39	< 0.01
	14	4765.45	0.87	18	19	3080.49	0.99
9	10	-344.78	1		20	3770.27	0.99
	11	988.4	1		21	-4202.09	0.97
	12	209.01	1		22	-4076.13	0.98
	13	1486.98	1	19	20	689.79	1

14	2949.73	0.99		21	-7282.58	0.18
15	4682.74	0.88		22	-7156.62	0.21
			20	21	-7972.38	0.08
				22	-7846.41	0.09
			21	22	125.96	1
